# Effect of Femtosecond Laser Processing Parameters on the Ablation Microgrooves of RB-SiC Composites

**DOI:** 10.3390/ma16062536

**Published:** 2023-03-22

**Authors:** Feng Yang, Renke Kang, Hongbin Ma, Guangyi Ma, Dongjiang Wu, Zhigang Dong

**Affiliations:** Key Laboratory for Precision and Non-Traditional Machining Technology of Ministry of Education, Dalian University of Technology, Dalian 116024, China

**Keywords:** RB-SiC composites, femtosecond laser, microgroove, laser processing parameter, ablation rate (AR)

## Abstract

Because of the high hardness, brittleness, and anisotropy of reaction-bonded silicon carbide composites (RB-SiC), it is challenging to process high-quality textures on their surfaces. With the advantages of high processing accuracy and low processing damage, femtosecond laser processing is the preferred technology for the precision processing of difficult-to-process materials. The present work used a femtosecond laser with a linear scanning path and a spot diameter of 18 µm to process microgrooves on RB-SiC. The influence of different processing parameters on the microgroove profile, dimensions, and ablation rate (AR) was investigated. The ablation width *W*_a_ and average ablation depth *D*_a_ of microgrooves were evaluated, and the various patterns of varying processing parameters were obtained. A model for *W*_a_ prediction was developed based on the laser fluence within the finite length (*F*_L_). As a result, the experimental values were distributed near the prediction curve with a maximum error of 20.4%, showing an upward trend of gradually decreasing increments. For a single pass, the AR value was mainly determined by the laser energy, which could reach the scale of 10^6^ μm^3^/s when the laser energy was greater than 50 μJ. For multiple passes, the AR value decreased as the number of passes increased and it finally stabilized. The above research will provide theoretical and experimental support for the high-quality and efficient processing of RB-SiC surface textures.

## 1. Introduction

Reaction-bonded silicon carbide composites (RB-SiC) consist of SiC grains with a Si matrix as a two-phase composite. RB-SiC is an advanced ceramic for its outstanding mechanical and thermal properties in a wide range of applications in aerospace, space optics, and nuclear energy [1,2,3]. The surface texturing technique effectively improved the service performance of RB-SiC components [4]. Mechanical machining and laser ablation have been successfully applied to generate surface patterns, but processing damage such as microcracks, subsurface damage, and heat-affected zones still exist [5,6,7]. The distribution of the two phases and the bonding force [8] in the particle-reinforced composite are changed by processing damage, which reduces the overall performance of the processed sample.

Femtosecond lasers promise high-precision processing of surface textures thanks to their ultra-short pulses, ultra-high laser energy density, and virtually no heat-affected zone [9,10]. Chen [11] developed a model for single-pulse energy ablation of single-crystal silicon by a femtosecond laser, which can accurately predict the geometric mechanism of ablation craters. The femtosecond laser micromachined the grooves on silicon wafers at different laser energies, translation speeds, the number of passes, and the effect of processing parameters on the groove depth and ablation rate were analyzed [12]. Zhang et al. [13] studied the multiscale evolution of V-grooves under femtosecond laser irradiation, analyzed the evolution mechanism, and established a self-organization model. The report by Deng et al. [14] analyzed the formation of microchannels and evaluated the effect of the laser processing parameters on the surface morphology and geometry systematically. The above studies investigated the ablation mechanism, profile, and morphology of single-crystal SiC. These provided theoretical support and analytical methods for the microstructural evolution of multiphase materials under femtosecond laser irradiation.

However, there are few reports on femtosecond laser processing RB-SiC composites. The physicochemical differences between SiC and Si make the femtosecond laser interaction with RB-SiC more complex. Chen et al. [15] performed selective polishing of RB-SiC by a femtosecond laser based on the ablation threshold difference between SiC and Si. It was found that when the optimized parameters were located between the ablation thresholds of the two phases, the roughness was reduced from 35 nm to about 11 nm, while maintaining a high polishing efficiency. Femtosecond laser ablation of microgrooves is required for high-quality and efficient processing of textured structures on RB-SiC surfaces, which consist of multiple microgrooves. Nevertheless, the information on femtosecond laser processing of RB-SiC microgrooves is insufficient. More detailed studies on the effect law and prediction model of different parameters on the geometry of microgrooves are needed to provide theory and data support for surface texture processing using a femtosecond laser.

This paper used a femtosecond laser to ablate microgrooves on RB-SiC samples. Different laser energies, scanning speeds, and number of passes in ablation experiments were conducted to systematically investigate the variation patterns of the microgroove width, average ablation depth, and AR. At the same time, a prediction model for microgroove geometry was established, which provided good support for the preferential selection of the process parameters.

## 2. Materials and Methods

**Figure 1:**
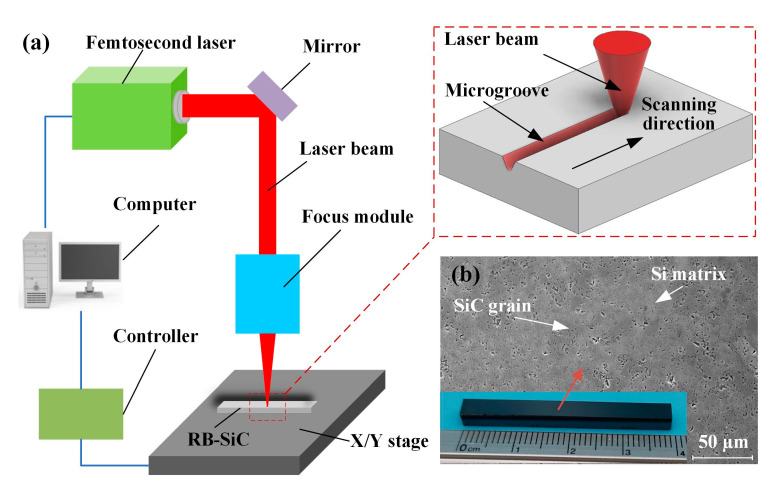
Schematic diagram of femtosecond laser system and materials: (**a**) schematic illustration of the laser system and (**b**) SEM image of RB-SiC.

As shown in Figure 1a, the femtosecond laser system consisted of a laser, a focus module, and an X/Y stage. The Gaussian laser beam emitted by the femtosecond laser (HR-Femto-IR-200-40, Wuhan Huaray Precision Laser Co., Ltd., Wuhan, China) was focused using a focusing module with a spot diameter of approximately 18 μm (focal length *f* = 60 mm). Moving the X/Y stage to achieve scanning of the RB-SiC sample (40 mm × 5 mm × 3 mm), the spot passed through the RB-SiC surface and formed a microgroove. RB-SiC was produced by infiltrating a mixture of silicon carbide grains and carbon into liquid silicon and sintering it by reaction bonding at high temperatures. The incomplete reaction resulted in residual Si in RB-SiC, consisting of SiC grains and Si matrix (Figure 1b). Before femtosecond laser scanning ablation, the RB-SiC was ground and polished to a surface roughness of less than Sa 20 nm using diamond abrasive paper of different grain sizes, and was then rinsed with ethanol in an ultrasonic cleaner for 10 min. After drying, the sample was subjected to a laser scanning ablation experiment. The parameters of the femtosecond laser and processing parameters used in the experiment are shown in Table 1. Scanning ablation experiments with different laser energies, scanning speeds, and number of passes were designed to analyze the profiles and their variation patterns at each parameter to provide a preferred range of parameters for process optimization. All of the scanning ablations were performed in a gas-free, protected environment at room temperature.

After the microgrooves of the RB-SiC sample were processed, their 3D topography was photographed using an optical microscope (Olympus, OLS4000). In addition, an electron microscope (FESEM, HITACHI SU5000) was used to detect the surface morphology of the microgrooves. For each microgroove, the width was measured in three different parts of the microgroove, and the average value of the whole microgroove was obtained, which was the measurement result of the microgroove width *W*_a_. The same method was used for volume measurements to obtain the ablation volume of the microgroove *V*.

## 3. Prediction Model of Ablation Width

**Figure 2:**
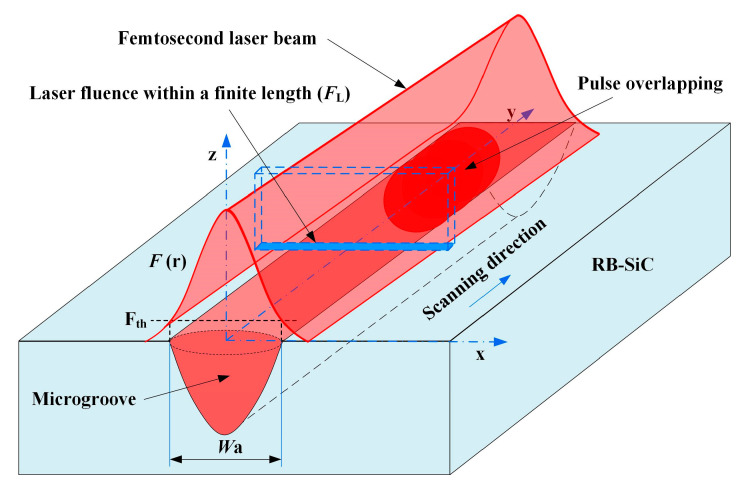
Schematic diagram of laser-ablated microgroove and the spatial distribution of laser fluence.

A schematic of RB-SiC ablated by femtosecond laser scanning is illustrated in Figure 2. The Gaussian distribution of the laser beam moves along the scanning direction to form a Gaussian laser beam. When the laser beam fluence exceeded the ablation threshold, F_th_, a near V-shaped microgroove was produced on the surface of RB-SiC [16]. The laser beam was discretized along the scanning direction to investigate the coupling effect of laser energy and scanning speed on the ablation microgroove. The laser fluence within the finite length (*F*_L_) was used instead of the above two parameters to analyze the variation pattern of the ablation width. In our regimes, *F*_L_ was proportional to the frequency (*f*) and laser energy (*E*_p_) and inversely proportional to the scanning speed (*v*) and the beam diameter (2*ω*), and can be expressed as Equation (1) [17].

(1) $$F_{L}=\frac{E_{p}f}{2\omega v}$$

**Figure 3:**
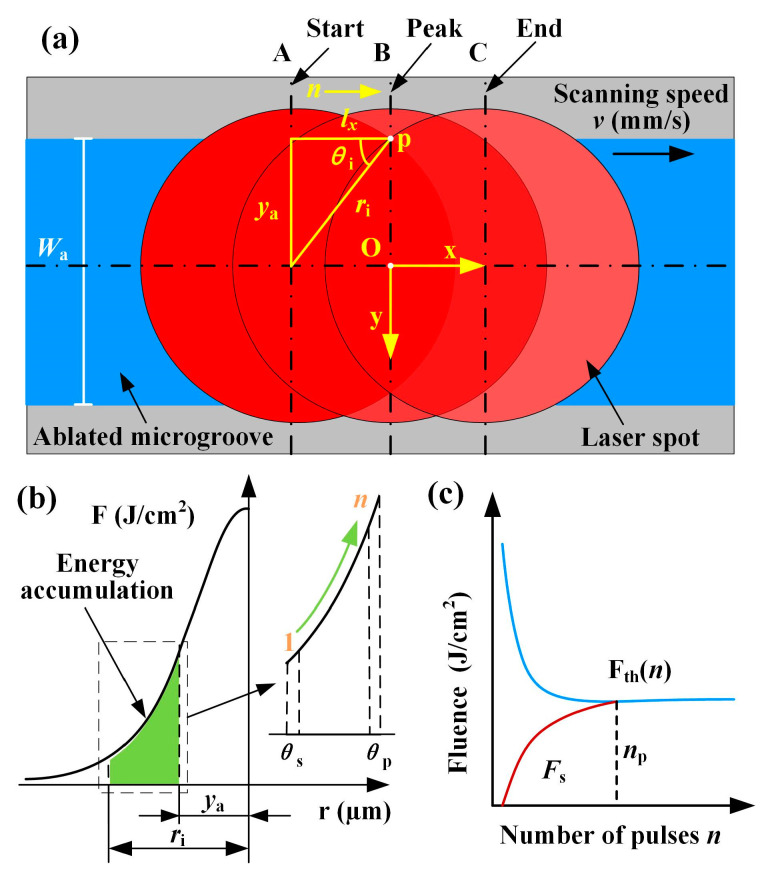
Schematic diagram of laser spot trajectory and energy accumulation. (**a**) The trajectory of the pulsed laser spot. (**b**) Schematic of energy accumulation of multiple pulses. (**c**) Effect of the number of pulses on the fluence accumulation and ablation threshold.

After the cumulative effect of multiple pulses of the femtosecond laser, the material in the scanned region was ablated, as shown in Figure 3. In Figure 3a, an arbitrary point p at the edge of the microgroove was taken, and the laser fluence accumulated at this point was calculated. In the scanned region, the laser spot needed to pass through stages A, B, and C along the x-direction. A is the starting stage when the laser spot started to act on point p. B is the peak stage, where the laser spot at point p had the highest laser fluence. Then, the spot moved from point p to stage C, and the cumulative effect of the pulsed laser ends. Stages A and C were symmetrical about stage B, which means that the laser fluence acting on point p was the same at the start and end stages. So, it was only necessary to analyze the energy accumulation process from the start stage A to the peak stage B.

The distance between stages A and B was set as *l*_x_. The number of pulses *n* accumulated between them was calculated as shown in Equation (2). As depicted in Figure 3b, the initial angle of stage A is *θ*_s_, the peak angle of stage B is *θ*_p_, and *θ*_i_ is the angle of *i* th spot in the scanning process, which is calculated as shown in Equation (3). The Gaussian distributed laser fluence decreased with increasing the distance from the spot center point so that the fluence was minimum at the angle of *θ*_s_ and maximum at the angle of *θ*_p_. According to the distance *r*_i_ from the center of any laser spot to point p, the laser fluence at point p could be deduced according to Equation (4), and ω was the irradiation radius of the laser spot on the sample surface. Based on Equations (2)–(4), the total laser fluence accumulated in a scanned region could be calculated as *F*_s_ (Equation (5)), where *k* is the accumulation factor.

(2) $$n=\frac{l_{x}f}{v}=\frac{y_{a}f}{\tan(\theta_{s})v}$$

(3) $$\theta_{i}=\theta_{s}+\frac{\left(\theta_{p}-\theta_{s}\right)}{n-1}(i-1)$$

(4) $$F_{i}=\frac{2E_{p}}{\pi \omega^{2}}\exp(-\frac{2r_{i}{}^{2}}{\omega^{2}})$$

(5) $$F_{s}=\sum_{i=1}^{n}F_{i}=\sum_{i=1}^{n}\frac{4kF_{L}v}{f\pi \omega }\exp\left(\frac{-2n^{2}v^{2}\tan^{2}\left(\theta_{s}\right)}{\omega^{2}f^{2}\sin^{2}\left(\theta_{s}+\frac{\left(\theta_{p}-\theta_{s}\right)}{n-1}(i-1)\right)}\right)$$

Considering the incubation effect of multiple pulse ablation materials, the change law of the multi-pulse ablation threshold F_th_(*n*) with the number of pulses *n* can be described as follows [18]:

(6) $$F_{\mathrm{th}}\left(n\right)=F_{\mathrm{th}}\left(\infty \right)+\left[F_{\mathrm{th}}-F_{\mathrm{th}}\left(\infty \right)\right]\times e^{-\alpha \left(n-1\right)}$$

F_th_ is the single pulse threshold fluence and *α* is the incubation coefficient. When *n* increases, the F_th_(*n*) is approximately equal to the minimum fluence F_th_(∞), and a lower laser fluence is required to achieve ablation of the material. Ablation occurred when the accumulated laser fluence at point p reached the multi-pulse ablation threshold within the A-B stage of the scanned region, where *F*_s_ = F_th_(*n*) (Figure 3c). Point p was the boundary position of the ablated microgroove, and the ablation width was calculated as two times the distance from this point to the centerline. A set of microgrooves with a sure *v* of different laser energies was chosen, and F_th_(*n*) could be found by fitting the data. It was known that *θ*_p_ is 90°and *θ*_s_ was taken as a 30°. The coefficient *k* was obtained by choosing four microgrooves corresponding to different scanning speeds. Based on the 4 *k* values, the number of pulses at different *F*_L_ was calculated, and then the four group widths were calculated according to formula *W*_a_ = 2*n* × tan(*θ*_s_) × *v*/*f*. The data were fitted, the average values of the coefficients were selected as the equation coefficients, and then the *W*_a_ prediction model corresponding to different *F*_L_ was developed.

## 4. Results and Discussion

### 4.1. Effects of Processing Parameters on Microgroove Width

#### 4.1.1. Effects of Laser Energy and Scanning Speed on Microgroove Width

**Figure 4:**
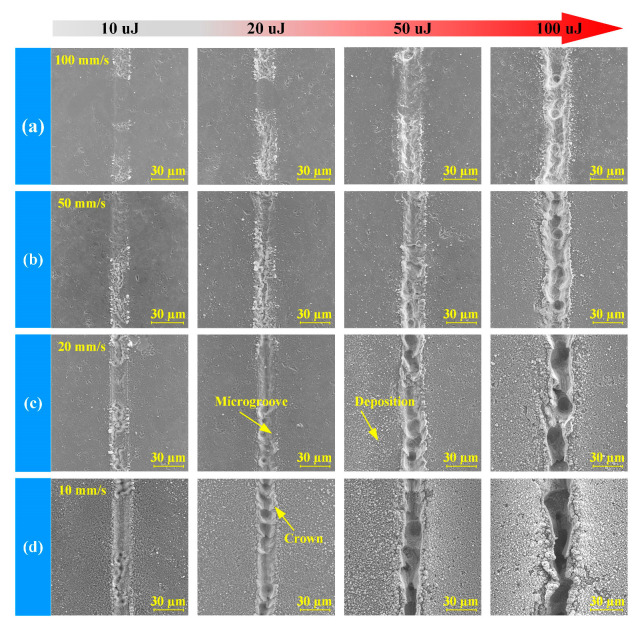
SEM image of different scanning speeds. (**a**–**d**) From 100 mm/s to 10 mm/s.

Figure 4 shows the SEM images with different laser energies and scanning speeds for one pass. After the femtosecond laser scan, a microgroove with a width of about 25 μm was produced on the RB-SiC surface. Many deposits were generated on both sides of the microgroove, produced by the sputtering deposition of products during the ablation process. With the increase in laser energy and the decrease in scanning speed, the ablative topography quality of the microgroove gradually deteriorated, and large areas of deposits and crowns appeared.

**Figure 5:**
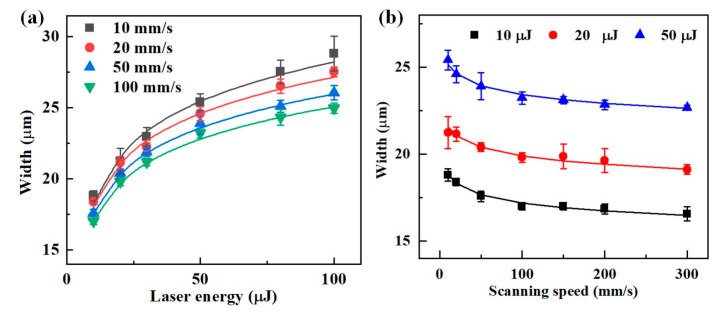
Effects of parameters on width. (**a**) The width varies with laser energy. (**b**) The width varies with scanning speed.

Figure 5 illustrates the variation of *W*_a_ with laser energy and scanning speed. As shown in Figure 5a, the microgroove width increased with *E*_p_ from 10 μJ to 100 μJ at different scanning speeds, and the rate of increase first increased and then decreased. Because of the Gaussian distribution of the laser, the laser fluence decreased with the increase in distance from the center point. At a lower *E*_p_, the fluence at the edge of the spot was lower than the ablation threshold of the material, resulting in a lower *W*_a_. When the *E*_p_ input was higher, the fluence area to reach the material ablation threshold was more extensive, and a wider microgroove could be obtained. However, because of the high *E*_p_, the loss of energy increased, resulting in a slowdown in the increase of the ablation width. The ablation width was measured for a scanning speed from 10 mm/s to 300 mm/s, which showed a decreasing trend with an increase in scanning speed (Figure 5b). The increase in scanning speed decreased the laser spot overlap rate, resulting in less energy absorbed in the irradiated region of the RB-SiC surface, allowing for a reduction in the ablation width. The results showed that at the same laser energy, there was little effect on the microgroove width when the scanning speed exceeded 100 mm/s.

**Figure 6:**
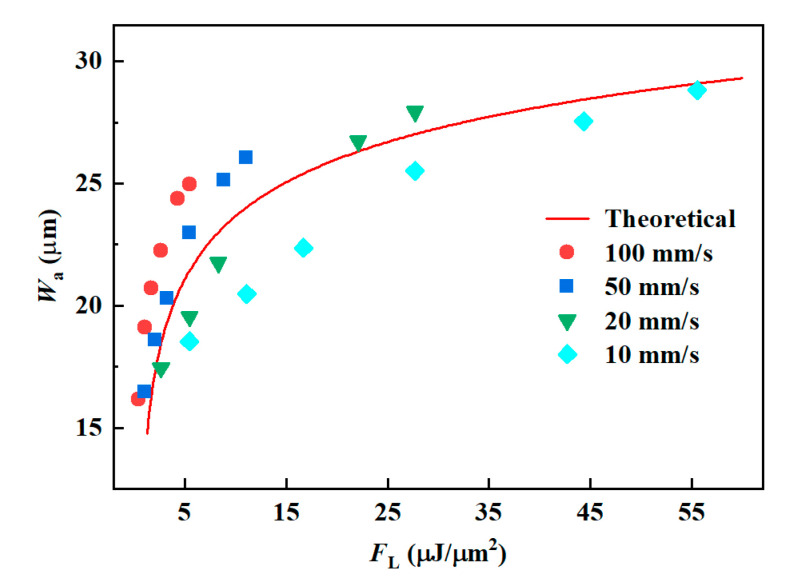
The *W*_a_ of the microgroove changes with the *F*_L_.

Table 2 shows the experimental parameters and the corresponding *F*_L_ values, which increased with the laser energy and decreased with the scan speed. The predicted results of *W*_a_ are shown in Figure 6, with the experimental data distributed around the prediction curve. There were several data deviations from the curve on the upper side of the curve when *v* (≥50 mm/s) and *E*_p_ (≥80 μJ) were high. When *F*_L_ = 2.78 μJ/μm^2^, the theoretical value was 22.25 μm, the experimental value was 18.47 μm, and the prediction error was as high as 20.5%. At this point, the thermal effect increased due to the enormous laser energy, making the ablation width slightly larger than the predicted value. The maximum deviation error below the curve was 14.7 when *F*_L_ = 11.11 μJ/μm^2^. At slower scanning speeds, the material was removed mainly in photothermal heat, leaving the ablation products to recondense at the edges of the microgrooves, so their widths were less than predicted.

#### 4.1.2. Effects of the Number of Passes on Microgroove Width

**Figure 7:**
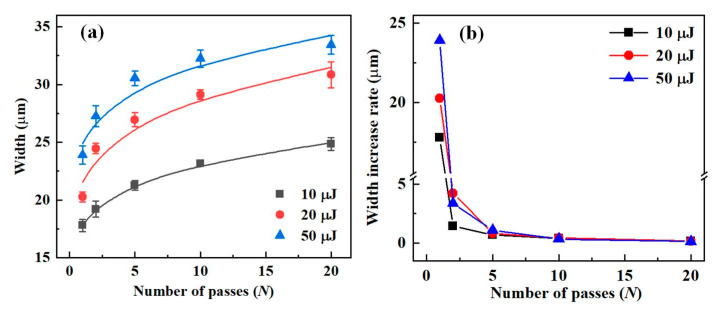
The width of the microgroove relative to *N* at a scanning speed of 50 mm/s. (**a**) The width and (**b**) width increase rate vary with the number of passes.

When the focused laser spot delivered multiple passes over the RB-SiC surface, it could be used to produce microgrooves with more significant material removal. Figure 7 shows the ablation width at a different number of passes for a scanning speed of 50 mm/s and laser energies of 10 μJ, 20 μJ, and 50 μJ, respectively. In Figure 7a, the width of the microgroove increased with the number of passes (*N* from 1 to 20), but the increase diminished. The variation in the width increase rate with *N* is shown in Figure 7b, showing an exponential decay followed by stabilization. At the three laser energies, the width increase rates were about 20 μm and 4 μm for one and two passes, respectively. When the number of passes exceeded 2, the width increase rate was less than 1 μm. The reason was that *F*_L_ mainly determined the ablation width of the microgroove, and when *F*_L_ was constant, increasing the number of passes had a less significant effect on the microgroove width.

### 4.2. Effects of Processing Parameters on Microgroove Depth

#### 4.2.1. Effects of Laser Energy and Scanning Speed on Microgroove Depth

**Figure 8:**
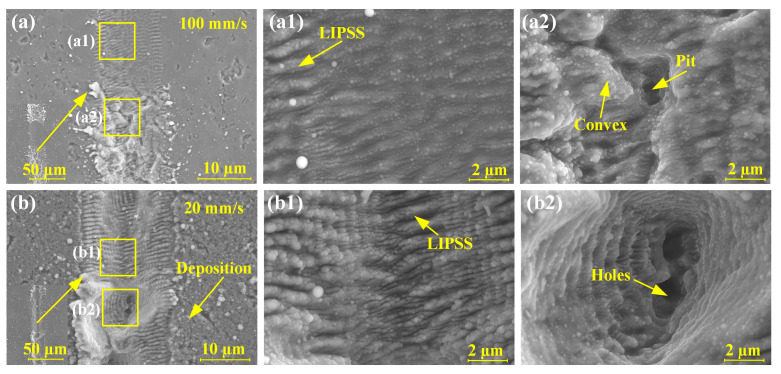
Microgroove morphology for different scanning speeds at a laser energy of 20 μJ: (**a**) 100 mm/s; (**a**1) Enlarged view of SiC grain region in Figure 8a; (**a**2) Enlarged view of Si matrix region in Figure 8a; (**b**) 20 mm/s; (**b**1) Enlarged view of SiC grain region in Figure 8b; (**b**2) Enlarged view of Si matrix region in Figure 8b.

The focused femtosecond laser spot passing through the RB-SiC surface could produce uneven microgrooves, as shown in Figure 8. For microgrooves after ablation with a scanning speed of 100 mm/s, laser-induced periodic surface structures (LIPSS) appeared in the SiC grain region (Figure 8a1), convex and pits appeared in the Si matrix region (Figure 8a2). When the scanning speed was 20 mm/s (Figure 8b), the difference in ablation morphology between the SiC grains and Si matrix regions was more obvious. In this case, the depth of LIPSS in the SiC grain region increased with a V-shape, and deeper holes appeared in the Si matrix region. The different physicochemical nature of SiC and Si presented different removal amounts under the same laser processing parameters, resulting in significant differences in the ablation morphology of both regions.

**Figure 9:**
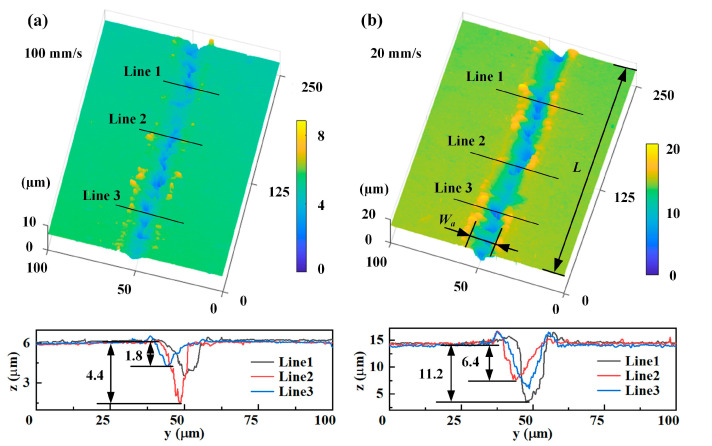
Three-dimensional confocal images and cross-sections of microgrooves at different scanning speeds: (**a**) 100 mm/s and (**b**) 20 mm/s.

Figure 9 shows the 3D confocal images and cross-sections for different scanning speeds with a laser energy of 20 μJ. In Figure 9a,b, three cross-sections of the microgrooves were chosen, where the different amounts of ablation of the SiC grains and the Si matrix resulted in poor uniformity of the groove bottom profile. Therefore, using the ablation depth to measure the material removal in the depth direction was not appropriate, so the average ablation depth was proposed. The average ablation depth *D*_a_ was calculated using the ablation volume (*V*) divided by the ablation area of the microgroove, as shown in Equation (6).

(7) $$D_{a}=\frac{V}{W_{a}L}$$

**Figure 10:**
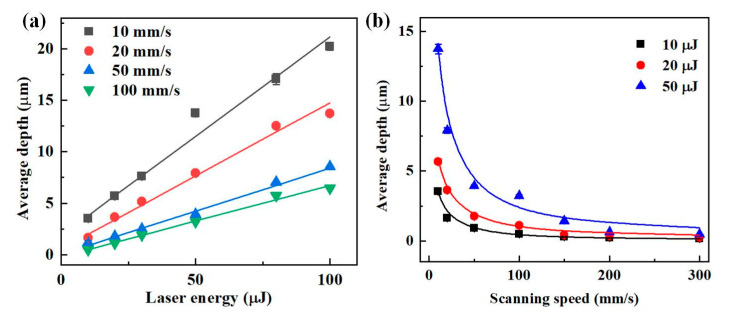
Effects of parameters on average depth. (**a**) The average depth is a function of laser energy. (**b**) The average depth is a function of the scanning speed.

**Figure 11:**
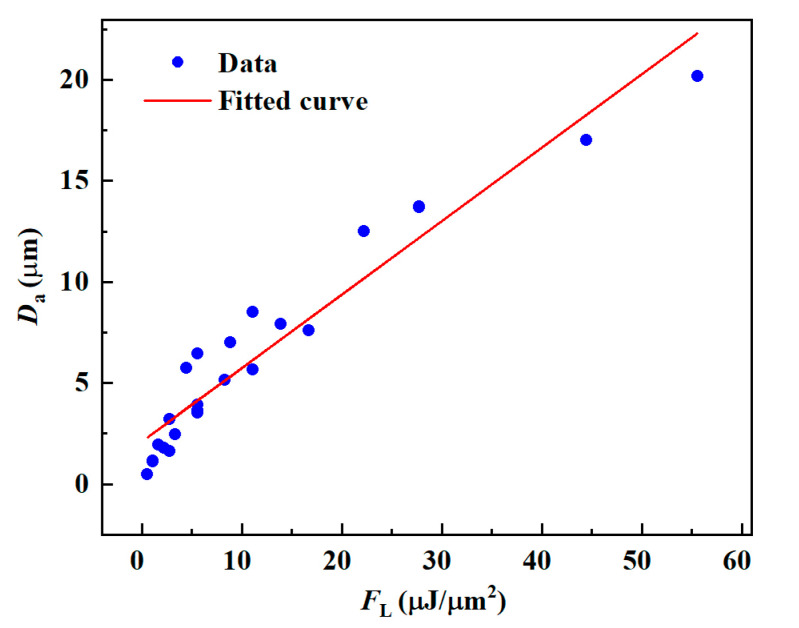
The average ablation depth *D*_a_ of the microgroove changes with the *F*_L_.

Figure 10 represents the average ablation depth of the microgrooves at different laser energies and scanning speeds. As shown in Figure 10a, the *D*_a_ of the microgroove increased linearly when increasing the laser energy for the same scanning speed. The increment increased from 0.07 μm/μJ to 0.19 μm/μJ when the scanning speed decreased from 100 mm/s to 10 mm/s within the same laser energy interval, showing the change law that the increment of *D*_a_ increased with the lower scanning speed. *D*_a_ decreased with increasing the scanning speed in Figure 10b and finally stabilized (about 0.4 μm). The high spot overlap rate at lower scanning speeds had a significant thermal effect, resulting in a high material removal and a considerable average ablation depth. At higher scanning speeds, the spot overlap rate was low, and less energy was deposited onto the target, resulting in a lower material removal. To a good approximation, *D*_a_ of the microgroove appeared to be linearly proportional to *F*_L_ (Figure 11). A larger finite-length laser fluence resulted in an immense average ablation depth.

#### 4.2.2. Effects of the Number of Passes on Microgroove Depth

**Figure 12:**
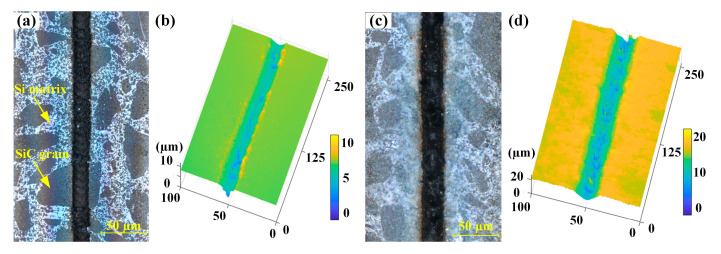
Microgroove morphology with laser energy of 10 μJ and scanning speed of 50 mm/s for the different number of passes: (**a**) 2D image and (**b**) 3D profile of a microgroove with 2 passes, and (**c**) 2D image and (**d**) 3D profile of a microgroove with 10 passes.

The focused femtosecond laser spot passing through the RB-SiC surface with multiple passes could produce deeper microgrooves. Figure 12 shows the morphology of the microgrooves at different passes for a laser energy of 10 μJ and a scanning speed of 50 mm/s. When comparing Figure 12a,c, the thickness of the deposits on both sides of the microgroove increased with the number of passes. The depth and unevenness of the microgroove bottom increased with the number of passes, as shown in Figure 12b,d. As the number of passes increased, the laser beam acted on the material several times, leading to more ablation, which in turn caused an increase in the microgroove ablation depth and the thickness of the ablation products deposited on both sides of the microgroove.

**Figure 13:**
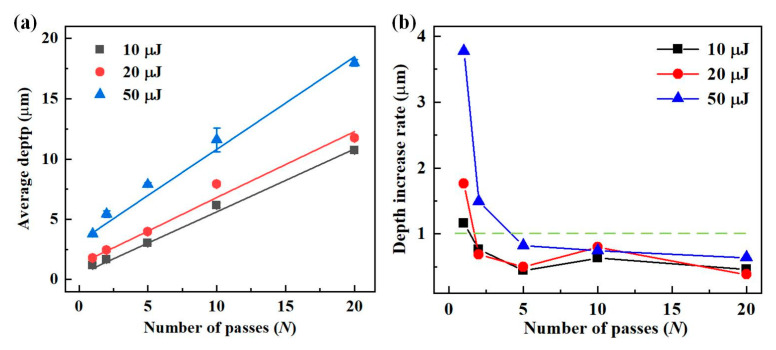
Effect of the number of passes on the depth of the microgrooves. (**a**) Variation in the average depth of microgrooves with the number of passes. (**b**) The depth increase rate in the microgroove varies depending on the number of passes.

Figure 13a shows *D*_a_ of the microgroove versus the number of passes at a scanning speed of 50 mm/s with three different laser energies. It can be seen that the average depth growth linearly increased as the number of passes of the femtosecond laser beam increased from 1 to 20. With increasing the laser energy from 10 μJ to 50 μJ, the slope of the fitted straight line of *D*_a_ versus the number of passes increased. As shown in Figure 13b, the depth increase rate decreased and stabilized as the number of passes increased. The values of the first pass ranged from 1 μm to 4 μm at three laser energies, while the depth increase rate for the subsequent passes was less than 1 μm. It was shown that the *D*_a_ of the microgroove did not change noticeably by only increasing the number of passes without shifting down the laser focus.

**Figure 14:**
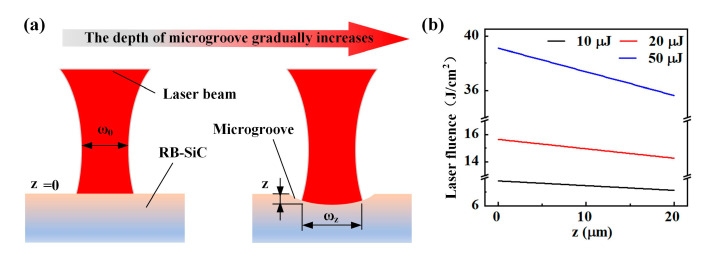
Effect of microgroove depth on the laser fluence. (**a**) Schematic diagram of the depth variation of the microgroove cross-section. (**b**) Variation in laser fluence with the depth of microgroove.

There were two main reasons for the above results, which were analyzed as follows. As shown in Figure 14a, the beam radius at different positions from the beam waist within the Rayleigh length ≈ 190 μm ($Z_{R}=\pi \omega_{0}^{2}/\lambda$) can be calculated by Equation (8) [19]. The laser fluence *F* varied with the radius corresponding to the depth *z*, which can be expressed as Equation (9).

(8) $$\omega =\omega_{0}\sqrt{1+{\left(\frac{\lambda z}{\pi \omega_{0}^{2}}\right)}^{2}}$$

(9) $$F=\frac{E_{p}}{\pi \omega^{2}}$$

Here, *ω*_0_ is the waist radius of the femtosecond laser beam, and *z* represents the depth of the ablation microgroove. After the first pass produced a microgroove, the laser was in a positive defocus state because the focal point did not move. As the depth increased with subsequent passes, the laser fluence at the defocused position gradually decreased, and the drop could reach 10% at a maximum depth of 20 μm (Figure 14b). Therefore, the first reason was that the laser fluence decreased due to the increased defocus, decreasing the depth increment gradually during subsequent passes. In the case of multiple passes, the laser beam was irradiated at the surface ablated by the previous pass for each pass after the first pass. The second reason is that the non-uniform surface at the bottom of the microgroove made the laser fluence transmission loss significant [20,21], leading to a decrease and eventual stabilization of the depth increase rate with an increase in the number of passes.

### 4.3. Ablation Rate of Microgroove

**Figure 15:**
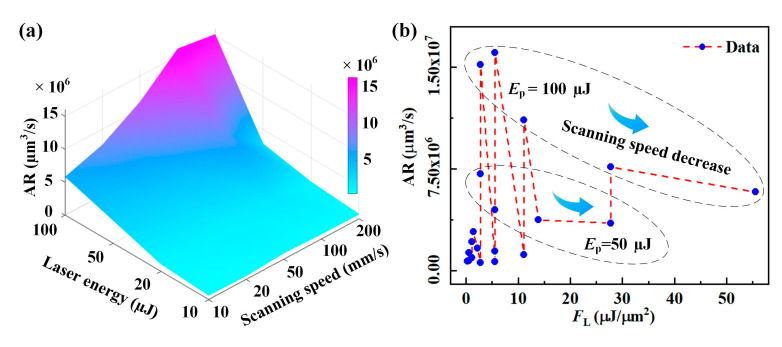
Effect of laser energy and scanning speed on AR. (**a**) Mapping of AR with laser energy and scanning speed. (**b**) The relationship between AR and *F*_L_.

The ablation rate (AR) is a crucial measure of the processing efficiency of femtosecond laser processing of RB-SiC microgrooves. The formula for AR for one pass ablation microgroove is shown in Equation (9). Figure 15a illustrates the mapping relationship of AR, which increased with the laser energy and scanning speed, where laser energy was the critical factor. Figure 15b shows the relationship between AR and *F*_L_ for one pass, and AR showed a trend of narrowing change amplitude with increasing *F*_L_. The results show that AR was high (the scale is 10^6^ μm^3^/s) when the laser energy was higher (50 μJ or 100 μJ) and decreased as the scanning speed decreased, indicating that the laser energy played a dominant role. Considering processing quality and efficiency, a laser energy of 50 μJ and a high scanning speed could be chosen to obtain a large AR.

(10) $$\mathrm{AR}=W_{a}D_{a}v$$

**Figure 16:**
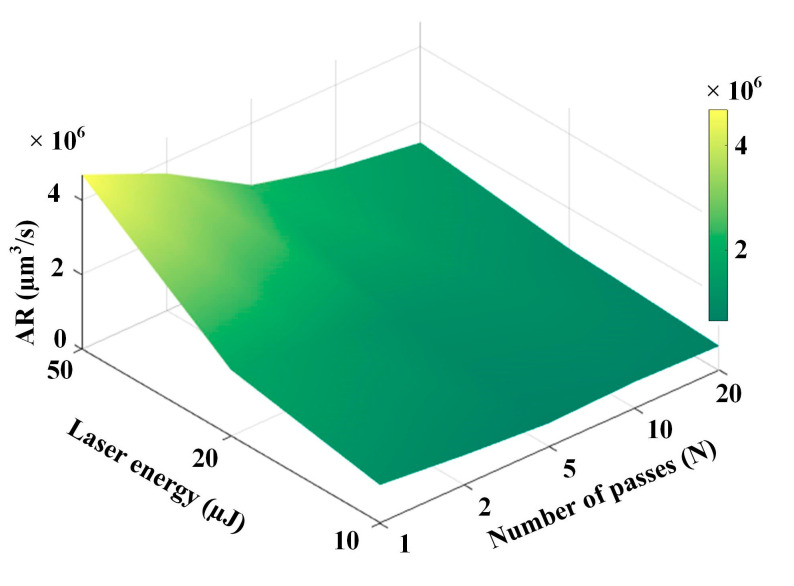
Mapping of AR with the number of passes and laser energy.

For the AR of the microgrooves after multiple pass ablation, the following equation is calculated:

(11) $$\mathrm{AR}(N)=\frac{V_{s}}{t_{s}}=\frac{\sum_{i=1}^{N}W_{a}\Delta D_{i}v}{N}$$

where vs. is the total removal volume after *N* passes, *t*_s_ is the time required for *N* passes, and Δ*D*_i_ is the average ablation depth of *i*th pass. As shown in Figure 16, AR was mapped by a different number of passes and laser energies. We found that the AR decreased when the number of passes increased, and its value stabilized when *N* exceeded 5. This suggests that the number of passes showed little effect on the AR of the microgroove. Therefore, high laser energy with few passes was appropriate for efficiently processing RB-SiC microgrooves.

## 5. Conclusions

Femtosecond laser ablation of microgrooves was performed for RB-SiC composites. Scanning ablation experiments investigated the variation in the geometry and surface morphology with different laser energies, scanning speeds, and number of passes. The findings, as well as the significance of this research, can be drawn as follows:

1. For microgroove ablation width *W*_a_ (about 25 μm), the laser energy is the main effect factor compared with the scan speed and the number of passes. Increasing the average ablation depth *D*_a_ of RB-SiC microgrooves was found to increase with the laser energy and the number of passes and decreasing with the increased scanning speed.
2. An energy accumulation analysis method based on the laser fluence *F*_L_ within the finite element length was proposed, a *W*_a_ prediction model was established, and the experimental results were distributed near the prediction curve with a maximum error of 20.4%. In the meantime, the average ablation depth *D*_a_ varied linearly with *F*_L_ at one pass.
3. The variation in AR of microgrooves under a single pass showed a narrowing trend with increasing *F*_L_, but the laser energy dominated the effect. The multi-pass AR value decreased as the passes count increased and finally stabilized. For suitable laser energy, a small number of passes (≤2) and a high scanning speed (≥50 mm/s) should be chosen to achieve a high processing efficiency.

## Figures and Tables

**Table 1:** The processing parameters applied for femtosecond laser scanning.

Characteristic	Value
Wavelength λ [nm]	1035
Pulse duration τ [fs]	350
Repetition rate *f* [kHz]	100
Laser energy *E*_p_ [μJ]	10–100
Scanning speed *v* [mm/s]	10–300
Number of passes *N*	1–20

**Table 2:** Experimental parameters and their corresponding *F*_L_.

Parameters	*F*_L_ (μJ/μm^2^)	Parameters	*F*_L_ (μJ/μm^2^)	Parameters	*F*_L_ (μJ/μm^2^)	Parameters	*F*_L_ (μJ/μm^2^)
10 μJ × 10 mm/s	5.56	10 μJ × 20 mm/s	2.78	10 μJ × 50 mm/s	1.11	10 μJ × 100 mm/s	0.56
20 μJ × 10 mm/s	11.11	20 μJ × 20 mm/s	5.56	20 μJ × 50 mm/s	2.22	20 μJ ×100 mm/s	1.11
30 μJ × 10 mm/s	16.67	30 μJ × 20 mm/s	8.33	30 μJ × 50 mm/s	3.33	30 μJ ×100 mm/s	1.67
50 μJ × 10 mm/s	27.78	50 μJ × 20 mm/s	13.89	50 μJ × 50 mm/s	5.56	50 μJ ×100 mm/s	2.78
80 μJ × 10 mm/s	44.44	80 μJ × 20 mm/s	22.22	80 μJ × 50 mm/s	8.89	80 μJ × 100 mm/s	4.44
100 μJ × 10 mm/s	55.56	100 μJ × 20 mm/s	27.78	100 μJ × 50 mm/s	11.11	100 μJ × 100 mm/s	5.56

## Data Availability

Not applicable.
